# Aged Lumbar Extension Strength of Chronic Low Back Pain in Korean Population of 10–80 Years

**DOI:** 10.18502/ijph.v49i10.4692

**Published:** 2020-10

**Authors:** Ji-Hoon CHO, Ki-Hyuk LEE, Seung-Taek LIM

**Affiliations:** 1. Department of Sport and Leisure Studies, Shingyeong University, Hwaseong, Republic of Korea; 2. Department of Sport Culture, Dongguk University, Seoul, Republic of Korea; 3. Institute of Sport Science, Kangwon National University, Gangwon-do, Republic of Korea; 4. Nasaret International Hospital, Incheon, Republic of Korea; 5. Waseda Institute for Sport Sciences, Waseda University, Saitama, Japan

**Keywords:** Lumbar, Extension strength, Age, Chronic low back pain

## Abstract

**Background:**

The purpose of this study was to find the basic data of medical and exercise therapy by indexing lumbar extension muscle strength of low back pain (LBP) patients.

**Methods:**

In this cross-sectional study, 3078 chronic LBP participants from The J hospital, Seoul, Republic of Korea, from 2003 to 2010 were enrolled. Maximum muscle strength was measured at maximum flexion angle and maximum extension angle according to range of motion (ROM) results. For each isometric test, participants were seated and secured in the MEDX (medx lumbar extension machine, Ocala, FL, USA) machine.

**Results:**

The relative ROM (*P*=0.012) differed significantly among the aged groups in all participants. In addition, mean of strength (*P*<0.001), maximal of strength (*P*<0.001), mean of strength %BW (*P*<0.001) and maximal of strength %BW (*P*<0.001) are significant differences in all participants. The results of multiple regression analysis was the ‘model A’, maximal of strength for 32.1% of the variance in weigh, body mass index and range of motion. In addition, ‘model B’ was 30.4%, ‘model C’ was 28.8%, ‘model D’ was 28.5%, ‘model E’ was 21.7%, and ‘model F’ was 23.5% of the variance in weigh, body mass index and range of motion.

**Conclusion:**

We found the three predictor (weight, BMI, and ROM) variables accounted for 32.1% of the variance in maximal of strength %BW, the highest in < 29 yr groups. Our data indicate the basic data of medical and exercise therapy by indexing lumbar extension muscle strength of LBP patients.

## Introduction

Low back pain (LBP) is a major health problem in Asian societies as well as in western societies today (1). Instability of the lumbar motor segment is considered important in chronic LBP (2). LBP is associated with aging, decreased physical activity, lumbar muscle mass, overall health or level of function, and other causes (3).

Parathyroid muscle dysfunction may be important for the pathogenesis of LBP (4, 5). Several of them focus on lumbar muscles, which are multifidus and paraspinal in LBP patients and the general population. The role of lumbar muscle multifidus and paraspinal in segment stiffness (6), control of the neutral region of the spinal segment (7), and the ability to stabilize the spine when challenging spinal stability (8). Multifidus and paraspinal muscle fatigue were greater in patients with chronic back pain compared to controls without LBP (9). In addition, atrophy examination of multifidus and paraspinal muscles was indicated in patients with chronic LBP (10). Therefore, an accurate assessment of muscle function may require examination of the back and spine muscles.

Lumbar extender, recently developed by MEDX (MEDX Lumbar Extender, Ocala, MA) to accurately measure the full range of lumbar extenders (11). It is a dynamometer that can be used to measure the isometric strength of muscles that extend the lumbar spine and provide dynamic and variable resistance exercise for the same muscles, proven to be a reliable and valid measurement and training tool (12, 13)

Thus, the presentation of lumbar extension muscle strength according to gender and age group of patients with LBP will be a measure of their lumbar muscle strength. It is necessary to provide data that is the basis of various pain relief and treatment of LBP patients, but it is very insufficient. Therefore, the purpose of this study was to find the basic data of medical and exercise therapy by indexing lumbar extension muscle strength of LBP patients.

## Materials and Methods

### Study participants

From January 2003 to December 2010, 3078 chronic LBP participants (male=1544, female=1534) were recruited from The J Hospital, Seoul, Republic of Korea (Table 1). All participants were complaining of nonspecific LBP without any structural or neuropsychological cause, for more than 3 months.

**Table 1:** The characteristic of the all participants

***Variable***	***Age (yr)***	***Height (cm)***	***Weight (kg)***	***BMI (kg/m^2^)***
< 29 yr (n = 369)	24.66 ± 3.30	171.4 ± 8.17	66.20 ± 13.22	22.37 ± 3.37
30–39 yr (n = 539)	34.58 ± 2.83	170.2 ± 7.92	66.94 ± 12.52	22.96 ± 3.11
40–49 yr (n = 571)	44.78 ± 2.80	166.0 ± 7.95	65.22 ± 11.12	23.54 ± 2.79
50–59 yr (n = 676)	54.32 ± 2.88	163.1 ± 8.01	63.21 ± 9.69	23.66 ± 2.66
60–69 yr (n = 595)	64.48 ± 2.71	160.6 ± 7.85	62.31 ± 8.48	24.12 ± 2.70
>70 yr (n = 328)	74.63 ± 4.17	159.6 ± 8.65	60.87 ± 9.24	23.84 ± 2.94

Values are mean (SD). BMI, body mass index

Exclusion criteria included a history of neurological, infectious, and systemic diseases, including cerebrovascular disease, spinal cord disease, spondylitis, cancer, rheumatologic disorders, and other chronic diseases that cause long-term immobilization. Participants who had undergone prior surgery for back pain were also excluded. The enrolment of study participants is shown in the flow char.

All the participants who agreed to participate in this study had the study explained to them to ensure a complete understanding of its purpose and the methods, in accordance with the ethical principles of the Declaration of Helsinki. The study passed Medical Ethics Committee review. The subjects also signed an informed consent form before participation.

### Measurements Lumbar extension strength

All participants completed isometric lumbar extension strength tests. Prior to testing, the participants completed 2–3 practice sessions to become familiar with the testing equipment and procedure. After the familiarization sessions, range of motion (ROM) was first measured for lumbar flexion before lumbar extension muscle strength test was performed considering all participants were LBP patients. Maximum muscle strength was measured at maximum flexion angle and maximum extension angle according to ROM results. For each isometric test, participants were seated and secured in the MEDX machine (medx lumbar extension machine, Ocala, FL, USA). Participants were then asked slowly to increase the lumber extension torque over 5 s. Once they reached the maximum torque, they were instructed to slowly reduce the torque. A 5-min rest period was provided between angle conditions. The results of the 2 tests were averaged and used as reference values. The isometric lumbar extension strength was measured using a MEDX lumbar extension machine at 7 angular positions of the upper body, which included 72°, 60°, 48°, 36°, 24°, 12°, and 0° of the trunk angle. Participants were positioned sitting upright in the equipment according to the procedure described in previous research. Previous studies showed that this equipment was highly reliable (r = 0.94–0.98) and valid for the quantification of isometric lumbar extension strength (14). The orders of angles were balanced across all participants.

### Statistical analysis

The SPSS version 25.0 for Windows (SPSS, Inc., Chicago, IL, USA) was used to perform all statistical evaluations. The lumbar extension strength was further analyzed for significant difference among the groups using a one-way ANOVA. Moreover, multiple regression analysis was used to examine the relationships between the maximal of strength and predictors (weight, body mass index, and range of motion). The age group differences were assessed using a post-hoc Bonferroni test if the ANOVA was significant. The coefficient of determination r^2^ was calculated for the regression equations. r^2^ represents the percentage of variance by the independent variables to predict a dependent variable. The relationships among variables were analyzed using Pearson’s correlation coefficients. Statistical significance was accepted at the 0.05 level. All variables are present as means and standard deviations.

## Results

### The lumbar extension strength according to gender and aged

The lumbar extension strength according to gender and aged are show in Table 2–4. The relative ROM (*P*=0.012) differed significantly among the aged groups in all participants. In addition, mean of strength (*P*<0.001), maximal of strength (*P*<0.001), mean of strength %BW (*P*<0.001) and maximal of strength %BW (*P*<0.001) were significant differences in all participants (Table 2)

**Table 2:** The lumbar extension strength of the all participants

***Variable***	***ROM (°)***	***Mean of Strength (lbs)***	***Maximal of Strength (lbs)***	***Mean of Strength (%BW)***	***Maximal of Strength (%BW)***
< 29 yr (n = 369)	68.20 ± 8.93	142.5 ± 60.3	190.6 ± 71.5	212.7 ± 73.8	283.9 ± 80.0
30–39 yr (n = 540)	68.93 ± 8.06	144.8 ± 61.0	191.2 ± 75.0	213.1 ± 70.9	280.7 ± 82.0
40–49 yr (n = 571)	69.04 ± 7.69	129.8 ± 53.8	172.5 ± 68.3	196.4 ± 64.9	259.9 ± 76.3
50–59 yr (n = 676)	69.87 ± 6.83	119.6 ± 48.2	157.5 ± 60.3	186.9 ± 61.3	245.5 ± 72.3
60–69 yr (n = 595)	69.41 ± 7.21	106.1 ± 44.3	143.7 ± 54.2	168.4 ± 59.5	227.8 ± 69.0
>70 yr (n = 328)	69.73 ± 6.97	92.1 ± 40.5	128.4 ± 67.3	149.3 ± 53.1	208.6 ± 61.7
*P*-value	0.012	< 0.001	< 0.001	< 0.001	< 0.001
Post-hoc	b	a, b, c, d, e, f, g, h, i, j, k, l, m, n			

Values are mean (SD). ROM, range of motion

a =significant between < 29 and 40–49,

b =significant between < 29 and 50–59,

c =significant between < 29 and 60–69,

d =significant between < 29 and >70,

e =significant between 30–39 and 40–49,

f =significant between 30–39 and 50–59,

g =significant between 30–39 and 60–69,

h =significant between 30–39 and >70,

i =significant between 40–49 and 50–59,

j =significant between 40–49 and 60–69,

k =significant between 40–49 and >70,

l =significant between 50–59 and 60–69,

m =significant between 50–59 and >70,

n =significant between 60–69 and >70

**Table 3:** The lumbar extension strength of the male subjects

***Variable***	***ROM (°)***	***Mean of Strength (lbs)***	***Maximal of Strength (lbs)***	***Mean of Strength (%BW)***	***Maximal of Strength (%BW)***
< 29 yr (n = 244)	68.07 ± 8.70	168.5 ± 55.3	224.4 ± 61.2	235.2 ± 73.9	311.9 ± 75.5
30–39 yr (n = 350)	69.15 ± 7.30	172.3 ± 55.8	226.9 ± 66.4	236.4 ± 69.2	310.4 ± 77.6
40–49 yr (n = 298)	68.09 ± 8.79	160.0 ± 53.3	214.8 ± 64.1	221.6 ± 67.7	296.7 ± 71.1
50–59 yr (n = 293)	69.50 ± 7.57	154.0 ± 47.3	202.9 ± 58.2	221.8 ± 61.1	291.8 ± 71.1
60–69 yr (n = 225)	69.65 ± 7.32	139.1 ± 47.0	186.0 ± 53.5	205.2 ± 62.1	274.3 ± 68.9
>70 yr (n = 135)	69.13 ± 7.95	113.5 ± 46.7	157.3 ± 55.8	171.1 ± 59.3	237.3 ± 67.8
*P*-value	0.078	< 0.001	< 0.001	< 0.001	< 0.001
Post-hoc		a, b, c, d, e, f, g, h, i, h, k, l	a, b, c, e, f, g, h, i, j, k, l	b, c, f, g, i, k, l	a, b, c, e, f, g, h, i, k, l

Values are mean (SD). ROM, range of motion

a =significant between < 29 and 50–59,

b =significant between < 29 and 60–69,

c =significant between < 29 and >70,

d =significant between 30–39 and 40–49,

e =significant between 30–39 and 50–59,

f =significant between 30–39 and 60–69,

g =significant between 30–39 and >70,

h =significant between 40–49 and 60–69,

i =significant between 40–49 and >70,

j =significant between 50–59 and 60–69,

k =significant between 50–59 and >70,

l =significant between 60–69 and >70

**Table 4:** The lumbar extension strength of the female subjects

***Variable***	***ROM (°)***	***Mean of Strength (lbs)***	***Maximal of Strength (lbs)***	***Mean of Strength (%BW)***	***Maximal of Strength (%BW)***
< 29 yr (n = 125)	68.45 ± 9.40	91.66 ± 29.22	124.6 ± 34.5	168.7 ± 50.4	229.3 ± 57.0
30–39 yr (n = 189)	68.51 ± 9.30	93.95 ± 29.37	125.1 ± 34.4	170.0 ± 51.2	225.8 ± 58.3
40–49 yr (n = 273)	70.07 ± 6.13	96.89 ± 29.59	126.4 ± 34.5	168.8 ± 48.7	219.8 ± 52.9
50–59 yr (n = 383)	70.15 ± 6.20	93.32 ± 28.23	122.7 ± 32.2	160.3 ± 46.1	210.2 ± 50.0
60–69 yr (n = 370)	69.28 ± 7.15	86.09 ± 28.50	118.0 ± 35.2	146.0 ± 45.1	199.5 ± 51.7
>70 yr (n = 193)	70.14 ± 6.19	77.34 ± 27.22	108.9 ± 32.6	134.2 ± 42.2	188.7 ± 48.2
*P*-value	0.024	< 0.001	< 0.001	< 0.001	< 0.001
Post-hoc		c, e, f, g, h, i, j, k	c, f, g, h, j, k	b, c, e, f, g, h, i, j	a, b, c, d, e, f, g, h, j

Values are mean (SD). ROM, range of motion

a =significant between < 29 and 50–59,

b =significant between < 29 and 60–69,

c =significant between < 29 and >70,

d =significant between 30–39 and 40–49,

e =significant between 30–39 and 50–59,

f =significant between 30–39 and 60–69,

g =significant between 30–39 and >70,

h =significant between 40–49 and 60–69,

i =significant between 40–49 and >70,

j =significant between 50–59 and 60–69,

k =significant between 50–59 and >70,

l =significant between 60–69 and >70

The relative mean of strength (*P*<0.001), maximal of strength (*P*<0.001), mean of strength %BW (*P*<0.001) and maximal of strength %BW (*P*<0.001) differed significantly among the aged groups in male participants. In addition, not significant differences in ROM (Table 3).

The relative ROM (*P*=0.024) differed significantly among the aged groups in all participants. In addition, mean of strength (*P*<0.001), maximal of strength (*P*<0.001), mean of strength %BW (*P*<0.001) and maximal of strength %BW (*P*<0.001) are significant differences in female participants (Table 4).

### Correlation coefficients with Maximal of strength (%BW)

Table 5 shows the correlation coefficients of among the variables. Negative correlation was found between maximal of strength %BW and age. Moreover, negative correlation was found between ROM and BMI, and ROM and weight. In addition, positive correlations were found between maximal of strength and BMI, weight and ROM.

**Table 5:** Pearson’s correlation coefficients

	***M_Strength (%BW)***	***Age***	***BMI***	***Weight***	***ROM***
M_Strength	-				
(%BW)					
Age	− .319^**^	-			
BMI	.048^**^	.171^**^	-		
Weight	.348^**^	− .177^**^	.745^**^	-	
ROM	.259^**^	.052^**^	− .062^**^	− .107^**^	-

M_Strength; maximal of strength, BMI; body mass index, ROM; range of motion

* *P*<.05,

** *P*<.01

### Multiple regression model estimating the association with Maximal of strength (%BW)

**Fig. 1:**
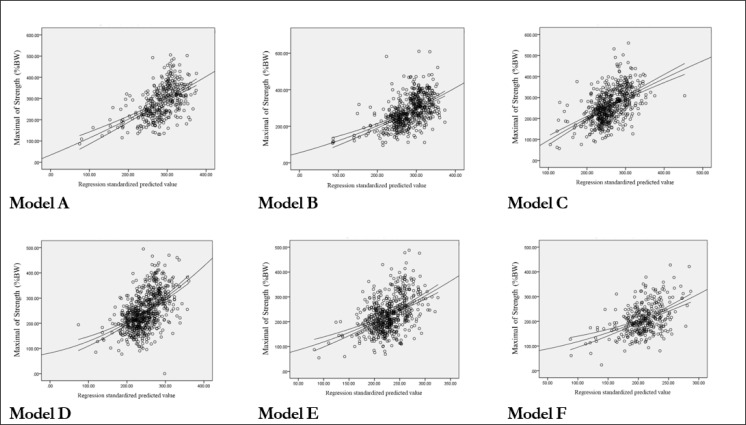
Scatter plot of the multiple regression analysis in enter model Predictors: (Constant), a = Weight, b = Body Mass Index (BMI), and c = Range of Motion (ROM). A. < 29 yr (n=369). Adjusted R^2^ = 0.321, (df = 3, F = 57.594, *P*< .001), a, β = .919 (*P*<.001), b, β = −.674 (*P*<.001), c, β = .422 (*P*<.001) B. 30~39 yr (n=539). Adjusted R^2^ = 0.304, (df = 3, F = 77.516, *P* < .001), a, β = .777 (*P*<.001), b, β = −.472 (*P*<.001), c, β = .363 (*P*<.001) C. 40~49 yr (n=571). Adjusted R^2^ = 0.288 (df = 3, F = 76.415, *P* < .001), a, β = .727 (*P*<.001), b, β = −.389 (*P*<.001), c, β = .350 (*P*<.001) D. 50~59 yr (n=676). Adjusted R^2^ = 0.285 (df = 3, F = 89.051, *P* < .001), a, β = .742 (*P*<.001), b, β = −.508 (*P*<.001), c, β = .271 (*P*<.001) E. 60~69 yr (n=595). Adjusted R^2^ = 0.217 (df = 3, F = 54.636, *P*< .001), a, β = .536 (*P*<.001), b, β = −.328 (*P*<.001), c, β = .264 (*P*<.001) F. > 70 yr (n=328). Adjusted R^2^ = 0.235 (df = 3, F = 33.095, *P* < .001), a, β = .488 (*P*<.001), b, β = −.210 (*P* = .002), c, β = .348 (*P*<.001)

The multiple regression analysis was carried out with intelligibility as the dependent variable and maximal of strength %BW. The scatter plot of the enter-mode analysis is showed in Fig. 1.

Based on the ‘model A’, maximal of strength for 32.1% of the variance in weigh, body mass index and ROM was seen. In addition, ‘model B’ is 30.4%, ‘model C’ is 28.8%, ‘model D’ was 28.5%, ‘model E’ was 21.7%, and ‘model F’ was 23.5% of the variance in weigh, body mass index and ROM.

## Discussion

The purpose of this study was to investigate the gender and age specific lumbar extension muscle strength of LBP patients. A decrease in maximum muscle strength with increasing age in male and female was found. In addition, negative correlation was found between maximal of strength and age, also positive correlation was found between maximal of strength and ROM.

Among the abdominal muscles, transverse abdominal multifidus and internal oblique muscles increase intraperitoneal pressure and contribute to the stability of the spine and pelvis (15). Any muscle that crosses the lumbar region has the potential to give stability to the lumbar spine (16). Therefore, the lumbar muscles of LBP patients are also important for spinal stabilization. Compared with other muscles close to the spinal cord, multifidus muscles contribute to two thirds of the increased stiffness by muscle contraction (6). In addition, lumbar spine and lumbar multifidus muscles were strongly associated with patient back pain (17). Disorders of spontaneous activation of multifidus and abdominal muscles have been reported in connection with recurrent or chronic LBP (18). In this study, we measured lumbar extension muscle strength in LBP patients. The importance of this finding is the decrease in maximal strength and increase in age in male and female. In addition, the three predictor (weight, BMI, and ROM) variables accounted for 32.1% of the variance in maximal of strength, the highest in < 29 yr’ groups. Whole body muscle strength was decreased caused by aging (19, 20). Strong and flexible muscles of the trunk play an important role in preventing many axial compressions and preventing sprains and chronic muscle tension (21). The strength of the trunk muscle is very important for improving the quality of life who chronic LBP patients ROM measurements are necessary because it is important to stabilize the pelvis and lower extremities in order to isolate lumbar muscles during accurate quantification of lumbar extension strength and during waist strength testing (11, 22). Thus, lumbar extension machines have been recently developed to accurately measure the range of locomotor extension strength, standardization of test and training positions (23). In this study, lumbar extension strength was measured according to the sex and age of LBP. In both males and females, the mean and maximal of muscle strength decreased with age, but there was a significant difference in ROM among females.

The differences in ROM by gender is that maintain the ability of chronic back pain to increase with muscle tension, concentric muscles to reduce blood circulation and balance body muscle tension.

The difference in ROM by gender is that chronic low back pain increases with muscle tension, concentric muscles reduce blood circulation, and maintains the ability to balance trunk muscle tension.

Chronic LBP increased with muscle tension, concentric muscles reduced blood circulation, and maintained the ability to balance trunk muscle tension might be affected to difference in ROM by gender (24). ROM in both the exercise group and the control group remained unchanged after the 10-week intervention period, with pain in a neutral posture rather than end-of-range symptoms (25). No differences in ROM were observed between patients with LBP and normal subjects (26). These results might be suggesting that neural networks can effect complex relationships between variables. However, we found positive correlation maximal of strength %BW and ROM. Eventually, patients with chronic LBP need improvement of lumbar muscle strength.

The present study had some limitations. We did not consider the type of LBP. However, type of LBP should be considered in future studies. Also further research is needed to assess the efficacy of this form of intervention in other LBP populations where the anatomic stability of the lumbar spine has been compromised.

## Conclusion

We examined the lumbar extension muscle strength according to gender and age group of patients with LBP. The relative mean of strength and maximal of strength differed significantly among the aged groups in both males and females. However, no differences were identified regarding the ROM in males. Our data might be used as a basic data of medical and exercise therapy by indexing lumbar extension muscle strength of LBP patients.

## Ethical considerations

Ethical issues (Including plagiarism, informed consent, misconduct, data fabrication and/or falsification, double publication and/or submission, redundancy, etc.) have been completely observed by the authors.

## References

[1] Johannsen F Remvig L Kryger P (1995). Exercises for chronic low back pain: a clinical trial. J Orthop Sports Phys Ther, 22(2):52–9. 7581431 10.2519/jospt.1995.22.2.52

[2] Friberg O (1987). Lumbar instability: a dynamic approach by traction-compression radiography. Spine (Phila Pa 1976), 12(2): 119–29. 2954216 10.1097/00007632-198703000-00007

[3] Manchikanti L Singh V Datta S (2009). Comprehensive review of epidemiology, scope, and impact of spinal pain. Pain Physician, 12:E35–70. 19668291

[4] Lima M Ferreira AS Reis FJJ (2018). Chronic low back pain and back muscle activity during functional tasks. Gait Posture, 61:250–6. 29413793 10.1016/j.gaitpost.2018.01.021

[5] Gizzi L Röhrle O Petzke F (2019). People with low back pain show reduced movement complexity during their most active daily tasks. Eur J Pain, 23(2):410–18. 30246275 10.1002/ejp.1318

[6] Wilke HJ Wolf S Claes LE (1995). Stability increase of the lumbar spine with different muscle groups. A biomechanical in vitro study. Spine (Phila Pa 1976), 20(2):192–8. 7716624 10.1097/00007632-199501150-00011

[7] Izzo R Guarnieri G Guglielmi G (2013). Biomechanics of the spine. Part I: spinal stability. Eur J Radiol, 82(1):118–26. 23088879 10.1016/j.ejrad.2012.07.024

[8] Kiefer A Shirazi-Adl A Parnianpour M (1997). Stability of the human spine in neutral postures. Eur Spine J, 6(1):45–53. 9093827 10.1007/BF01676574 PMC3454628

[9] Biedermann HJ Shanks GL Forrest WJ (1991). Power spectrum analyses of electromyographic activity. Discriminators in the differential assessment of patients with chronic low-back pain. Spine (Phila Pa 1976), 16(10):1179–84. 1836678

[10] Danneels LA Vanderstraeten GG Cambier DC (2000). CT imaging of trunk muscles in chronic low back pain patients and healthy control subjects. Eur Spine J, 9(4):266–72. 11261613 10.1007/s005860000190 PMC3611341

[11] Pollock ML Leggett SH Graves JE (1989). Effect of resistance training on lumbar extension strength. Am J Sports Med, 17(5):624–9. 2532867 10.1177/036354658901700506

[12] Weissenfels A Teschler M Willert S (2018). Effects of whole-body electromyostimulation on chronic nonspecific low back pain in adults: a randomized controlled study. J Pain Res, 11:1949–57. 30288089 10.2147/JPR.S164904 PMC6160275

[13] Shirley FR O’Connor P Robinson ME (1994). Comparison of lumbar range of motion using three measurement devices in patients with chronic low back pain. Spine (Phila Pa 1976), 19(7):779–83. 8202795 10.1097/00007632-199404000-00009

[14] Graves JE Pollock ML Carpenter DM (1990). Quantitative assessment of full range-of-motion isometric lumbar extension strength. Spine(Phila Pa 1976), 15(4):289–94. 2141187 10.1097/00007632-199004000-00008

[15] Richardson CA Snijders CJ Hides JA (2002). The relation between the transversus abdominis muscles, sacroiliac joint mechanics, and low back pain. Spine (Phila Pa 1976), 27(4):399–405. 11840107 10.1097/00007632-200202150-00015

[16] Bergmark A (1989). Stability of the lumbar spine. A study in mechanical engineering. Acta Orthop Scand Suppl, 230:1–54. 2658468 10.3109/17453678909154177

[17] Mengiardi B Schmid MR Boos N (2006). Fat content of lumbar paraspinal muscles in patients with chronic low back pain and in asymptomatic volunteers: quantification with MR spectroscopy. Radiology, 240(3): 786–92. 16926328 10.1148/radiol.2403050820

[18] Mannion AF Caporaso F Pulkovski N (2012). Spine stabilisation exercises in the treatment of chronic low back pain: a good clinical outcome is not associated with improved abdominal muscle function. Eur Spine J, 21(7):1301–10. 22270245 10.1007/s00586-012-2155-9 PMC3389103

[19] Fried LP Tangen CM Walston J (2001). Frailty in older adults: evidence for a phenotype. J Gerontol A Biol Sci Med Sci, 56(3):M146–56. 11253156 10.1093/gerona/56.3.m146

[20] Wessner B Liebensteiner M Nachbauer W (2019). Age-specific response of skeletal muscle extracellular matrix to acute resistance exercise: A pilot study. Eur J Sport Sci, 19(3):354–64. 30293527 10.1080/17461391.2018.1526974

[21] Liemohn W (1988). Flexibility and Muscular Strength. J Physical Education Recreation & Dance, 59:37–40.

[22] Smidt G Herring T Amundsen L (1983). Assessment of abdominal and back extensor function. A quantitative approach and results for chronic low-back patients. Spine (Phila Pa 1976), 8(2):211–9. 6222489 10.1097/00007632-198303000-00014

[23] Graves JE Pollock ML Foster D (1990). Effect of training frequency and specificity on isometric lumbar extension strength. Spine (Phila Pa 1976), 15(6):504–9. 2144914 10.1097/00007632-199006000-00014

[24] Hunter JB Critz JB (1971). Effect of training on plasma enzyme levels in man. J Appl Physiol, 31(1):20–3. 5556957 10.1152/jappl.1971.31.1.20

[25] O’Sullivan PB Phyty GD Twomey LT (1997). Evaluation of specific stabilizing exercise in the treatment of chronic low back pain with radiologic diagnosis of spondylolysis or spondylolisthesis. Spine (Phila Pa 1976), 22(24):2959–67. 9431633 10.1097/00007632-199712150-00020

[26] Marras WS Lavender SA Leurgans SE (1995). Biomechanical risk factors for occupationally related low back disorders. Ergonomics, 38(2):377–410. 7895740 10.1080/00140139508925111

